# Biodiversity of a boreal mire, including its hydrographic network (Shichengskoe mire, north-western Russia)

**DOI:** 10.3897/BDJ.9.e77615

**Published:** 2021-11-24

**Authors:** Dmitriy A. Philippov, Sergey G. Ermilov, Vera L. Zaytseva, Sergey V. Pestov, Eugeniy A. Kuzmin, Julia N. Shabalina, Alexey S. Sazhnev, Ksenya N. Ivicheva, Irina N. Sterlyagova, Mikhail M. Leonov, Margarita A. Boychuk, Andrey B. Czhobadze, Kristina I. Prokina, Mikhail V. Dulin, Omid Joharchi, Aleksey A. Shabunov, Olga S. Shiryaeva, Andrey N. Levashov, Aleksandra S. Komarova, Victoria V. Yurchenko

**Affiliations:** 1 Papanin Institute for Biology of Inland Waters Russian Academy of Sciences, Borok, Russia Papanin Institute for Biology of Inland Waters Russian Academy of Sciences Borok Russia; 2 Institute of Forest Science, Russian Academy of Sciences, Uspenskoe, Russia Institute of Forest Science, Russian Academy of Sciences Uspenskoe Russia; 3 Tyumen State University, Tyumen, Russia Tyumen State University Tyumen Russia; 4 Vologda Branch of the Russian Federal Research Institute of Fisheries and Oceanography, Vologda, Russia Vologda Branch of the Russian Federal Research Institute of Fisheries and Oceanography Vologda Russia; 5 Institute of Biology of Komi Scientific Centre of the Ural Branch of the Russian Academy of Sciences, Syktyvkar, Russia Institute of Biology of Komi Scientific Centre of the Ural Branch of the Russian Academy of Sciences Syktyvkar Russia; 6 Vyatka State University, Kirov, Russia Vyatka State University Kirov Russia; 7 Independent Researcher, Saint Petersburg, Russia Independent Researcher Saint Petersburg Russia; 8 Syktyvkar State University named after Pitirim Sorokin, Syktyvkar, Russia Syktyvkar State University named after Pitirim Sorokin Syktyvkar Russia; 9 Independent Researcher, Moscow, Russia Independent Researcher Moscow Russia; 10 Institute of Biology of Karelian Research Centre Russian Academy of Sciences, Petrozavodsk, Russia Institute of Biology of Karelian Research Centre Russian Academy of Sciences Petrozavodsk Russia; 11 Vologda State University, Vologda, Russia Vologda State University Vologda Russia; 12 Institute of Plant and Animal Ecology of the Ural Branch of the Russian Academy of Sciences, Yekaterinburg, Russia Institute of Plant and Animal Ecology of the Ural Branch of the Russian Academy of Sciences Yekaterinburg Russia; 13 Institution of Additional Education “Center of Creativity”, Vologda, Russia Institution of Additional Education “Center of Creativity” Vologda Russia

**Keywords:** Russia, Eastern Europe, Vologda Region, dataset, mire, wetland, in-mire water bodies, *Sphagnum*, occurrence, data paper, Red Data Book

## Abstract

**Background:**

The paper is based on the dataset whose purpose was to deliver, in the form of GBIF-mediated data, diverse materials on the biodiversity of a large mire, Shichengskoe mire (Vologda Region, north-western Russia), including its various mire sites and intra-mire water bodies. The dataset was based on our materials collected for two decades (from 2000 to 2021) in different parts and biotopes of the Shichengskoe mire and complemented by scarce data obtained previously by other researchers. The data contain materials on the diversity of Animalia (2886 occurrences), Bacteria (22), Chromista (256), Fungi (111), Plantae (2463) and Protozoa (131). Within the study period, the most detailed and long-term biodiversity studies were carried out for higher plants and invertebrates. On the other hand, the data on the composition of lichens, protozoa, algae, basidiomycetes, some groups of invertebrates and, to a lesser extent, lichens and vertebrates are far less comprehensive and require further substantial research efforts. The list includes occurrences from both the peatland (mire sites and mire margins different in typology) and the objects of the mire hydrographic network. In a standardised form, this article summarises both already published (mainly in Russian) and unpublished materials.

**New information:**

The paper summarises the results of long-term research on the biodiversity of a boreal mire, including its hydrographic network. A total of 5869 occurrences were included in the dataset published in the Global Biodiversity Information Facility (GBIF, gbif.org) for the first time. According to the GBIF taxonomic backbone, the dataset covers 1358 taxa, including 1250 lower-rank taxa (species, subspecies, varieties, forms) and 108 taxa identified to the genus level. Several species found in the Shichengskoe mire, mainly belonging to Bacteria, Chromista and Protozoa, have never been listed in GBIF for the territory of Russia before. The overwhelming majority of occurrences and identified species came from the territory of Shichengskiy Landscape Reserve. Due to our work, this Reserve is now the most studied regional reserve in the Vologda Region with respect to biodiversity. By the number of revealed species, it is close to two federal protected areas: Darwinskiy State Nature Biospheric Reserve and National Park "Russkiy Sever".

## Introduction

The first data on the biodiversity of the Shichengskoe mire were obtained during short visits of scientists from the Vologda State Pedagogical University to study the lakes in the region back in 1972 and to investigate the territory in order to create a new protected area in the Vologda Region back in 1986; the materials of these works were published in a very condensed form (Vorobyev et al. 1981, Bobrovskiy et al. 1993). By the beginning of the 21^st^ century, data on the biodiversity of Shichengskoe mire and surrounding area were very scarce (Vorobyev 2007, Philippov 2010).

In July-August 2000 and 2002, two field studies by Svetlana P. Bobrova with a group of secondary school students were carried out in Shichengskoe Lake, Polyanok Lake and Plakunovskoe Lake (Smirnov 2002, Evgrafova 2004).

Our studies of the Shichengskoe mire began in 2000 and continue to the present day. Between 2000 and 2003, field research was carried out by Dmitriy A. Philippov as part of his university graduate thesis supervised by Andrey N. Levashov. The graduate thesis entitled "Flora of the Shichengskiy Landscape Reserve and its analysis" contained data on 177 species of vascular plants. For several following years, the biodiversity studies of the Shichengskoe mire were fragmentary.

Since 2009, a purposeful collection of data on the composition and structure of various groups of living organisms in the Shichengskoe mire has begun. Mikhail V. Dulin took part in liverworts research in May 2009; vascular plants, fungi and lichens were investigated with the help of Victoria V. Yurchenko in early October 2009.

Significant impact to the studies of the Shichengskoe wetland occurred when the focus shifted to the hydrobiological studies of different types of intra-mire water objects (Philippov 2017). During vegetation seasons in 2012-2015, the primary attention was paid to zooplankton (Zaytseva et al. 2016, Zaytseva et al. 2017, Lobunicheva and Philippov 2017), zoobenthos (Ivicheva and Philippov 2013, Ivicheva and Philippov 2017), bacterio- and virioplankton (Stroynov and Philippov 2017a, Stroynov and Philippov 2017b) and phytoplankton. At that time, we summarised materials on vascular plants (Philippov 2015a, Bobroff et al. 2017), mosses (Philippov and Boychuk 2015), liverworts (Philippov and Dulin 2015) and birds (Philippov and Shabunov 2013) and studied plant-invertebrate interactions of *Utriculariaintermedia* (Zaytseva et al. 2014). In July 2012, *Sphagnum* mosses were sampled to study the testate amoebae composition (Philippov and Leonov 2017). In 2013, samples of terrestrial chortobionts (insects and spiders) were collected (Pestov and Philippov 2016, Pestov and Philippov 2021). In 2013 and 2014, an investigation of *Sphagnum* mosses growth rate in the Shichengskoe mire was conducted (Bengtsson et al. 2021). In 2015, samples of microalgae (Sterlyagova et al. 2016) and protozoa (Prokina et al. 2016, Prokina et al. 2017, Prokina and Philippov 2018, Prokina 2020) were collected. In 2014, 2016 and 2019, materials on mites inhabiting in mire sites with *Sphagnum* mosses were gathered (Minor et al. 2016, Minor et al. 2019). In August 2019, September 2020 and June 2021, samples of peat soils for metagenomic studies of prokaryotic diversity were collected (Ivanova et al. 2020, Dedysh et al. 2021). In 2019-2021, with the help of Aleksandra S. Komarova, aquatic and semi-aquatic insects (mainly Coleoptera) were collected (Sazhnev et al. 2019b). Field studies of lichens, fungi, fish and terrestrial vertebrates were always incidental. The materials were partly published as new regional findings of rare, new or interesting species: mosses and liverworts (Afonina et al. 2010, Dulin and Philippov 2010, Abolin et al. 2011, Sofronova et al. 2013, Sofronova et al. 2015, Sofronova et al. 2018, Sofronova et al. 2019), vascular plants (Philippov 2015c), algae (Kapustin et al. 2016a, Kapustin et al. 2016b, Vishnyakov and Philippov 2018), lichens (Czhobadze and Philippov 2015), insects (Ivicheva and Philippov 2015, Philippov 2015b, Philippov 2015d, Prokin et al. 2016, Sazhnev and Philippov 2017, Sazhnev et al. 2017, Sazhnev et al. 2019a, Sazhnev et al. 2020) and birds (Philippov and Shabunov 2014, Philippov 2016, Shabunov and Philippov 2018, Shabunov et al. 2019).

It is worth noting that we also obtained data on the hydrochemical composition of water (Philippov 2014, Philippov and Yurchenko 2020), microclimatic conditions (Philippov and Yurchenko 2019), heavy metal contamination of the mire surface (Shevchenko et al. 2018) and mercury contamination of peat (Udodenko and Philippov 2017).

Thus, over the past two decades, a significant amount of multifaceted materials on the biodiversity of the Shichengskoe mire and its hydrographic network has been accumulated, which we summarised in a GBIF dataset (Philippov et al. 2021).

## Project description

### Title

Biodiversity and conservation of mires of Nothern Russia

### Personnel

Dmitriy A. Philippov

## Sampling methods

### Study extent

The list of occurrences of different taxonomic and ecological groups of organisms inhabiting a large wetland in north-western Russia, the Shichengskoe mire, is presented. At the time we started our studies, only fragmentary data on the biodiversity of the Shichengskoe mire had been obtained (dated 1972, 1986, 2000 and 2002). Our work began in 2000 and continues to this day. The most detailed and long-term biodiversity studies were carried out for higher plants and terrestrial and aquatic invertebrates. The data on the composition of lichens, protozoa, algae, basidiomycetes, some groups of invertebrates (e.g. Collembola), lichens and vertebrates are far more scarce and require further substantial research efforts. The dataset includes species observations made both within the peat bog (mire sites and mire margins differed in typology) and in the mire hydrographic network, which we consider a structural element integral to the mire ecosystem (Philippov 2017). This dataset includes both published and unpublished materials.

### Sampling description

Biodiversity studies in the Shichengskoe mire were conducted from April to October, employing the route, reconnaissance and semi-stationary field approaches. Most microhabitats [a habitat which is of small or limited extent and which differs in character from some surrounding, more extensive habitat] were studied regularly during one or several vegetation seasons, but some were visited only once. The set of methods and techniques used in the field depended on both financial, time and logistical capabilities and the available specialists for specific taxonomic groups. We used a general approach to hydrobiological and ecological research of mires developed by the authors to study wetlands in Russia, described in the publication (Philippov et al. 2017).

### Quality control

The data were collected and identified by scientists from Papanin Institute for Biology of Inland Waters Russian Academy of Sciences, Tyumen State University, Vologda Branch of the Russian Federal Research Institute of Fisheries and Oceanography, Vologda State University, Institute of Biology of Komi Science Centre of the Ural Branch of the Russian Academy of Sciences, Syktyvkar State University named after Pitirim Sorokin, Institute of Biology of Karelian Research Centre of the Russian Academy of Sciences and the Institute of Plant and Animal Ecology of the Ural Branch of the Russian Academy of Sciences. The accuracy of identification of some samples was confirmed by the experts from Timiryazev Institute of Plant Physiology of the Russian Academy of Sciences.

### Step description

I. Research problem formulation.

II. Logistic issues resolution, including planning the location of routes, selection of water object, time and duration of work.

III. Field stage: obtaining samples and other original materials on the biodiversity of various components of the mire ecosystem.

(a) Macrophytes. In the field, pictures of plants and floristic lists were made, some species were collected in a herbarium (Philippov 2015a, Philippov and Boychuk 2015, Philippov and Dulin 2015, Bobroff et al. 2017); several hydrochemical parameters (water temperature, total dissolved solids, pH and electrical conductivity) were measured using portable devices. On the model sampling plots, relevés were made for mire sites different in microrelief (strings/ridges, hummocks, lawns, hollows, hollow-pools).

(b) Fungi. Basidiomycetes and lichens were studied on the way; as a rule, they were photographed and some samples were collected in the herbarium (Czhobadze and Philippov 2015).

(c) Algae. Samples were collected from the surface layer of water in several spots within the studied microhabitat using a plankton nylon net with a 20 μm pore diameter and a plastic sampler. Samples were fixed with 4% formalin (Kapustin et al. 2016b, Sterlyagova et al. 2016).

(d) Protozoa. Samples of heterotrophic flagellates and centrohelid heliozoans were collected in various microhabitats (water, upper peat or sediment layers, plants – by squeezing or washing off). Samples were collected in plastic tubes and transported to the laboratory at 4°C (Prokina et al. 2017, Prokina and Philippov 2018). To study testate amoebae diversity, *Sphagnum* mosses were collected in plastic tubes; the number of individual plants varied depending on different species growth densities (Philippov and Leonov 2017).

(e) Aquatic invertebrates. Zooplankton samples were collected at the model mire sites (lake, hollow-pool, fen strip, hollow and mire stream) by filtering water (5 to 50 litre) through a plankton net with 74 µm mesh. Samples were preserved with 4% formalin (Zaytseva et al. 2016, Zaytseva et al. 2017, Lobunicheva and Philippov 2017). Benthos invertebrates were collected at the model mire sites (lakes, fen strip and mire stream) with a bottom scraper (20 × 20 cm area). Each sample was washed through a 250 μm mesh nylon sieve, put in a plastic container and preserved in 40% formaldehyde. In mire streams and lakes, macrophyte-associated invertebrates were sampled; for that, water mosses clumps and aquatic plants with floating leaves were placed in plastic containers and preserved in 40% formaldehyde (Ivicheva and Philippov 2013, Ivicheva and Philippov 2017). The composition of aquatic, semi-aquatic and amphibiotic beetle communities was studied using trampling and sweeping procedures (Sazhnev and Philippov 2017, Sazhnev et al. 2019b, Sazhnev et al. 2020) and ethanol preservation of imagines and larvae.

(f) Terrestrial and soil invertebrates. The study of terrestrial insects and arachnids was carried out mainly on three model sites (fen strip, a ridge-hollow site and a mire stream valley at the mire margin) using a sweeping technique (30 sweeps in triplicate; diameter of the hoop 30 cm) ("Pollard walks") (Pestov and Philippov 2021). Manual collection of insects was performed outside the model sampling plots. Captured arthropods were euthanised with diethyl ether (Golub et al. 2021). Ticks were studied mainly in *Sphagnum*-dominated communities. Within the selected mire sites, samples were collected in microhabitats - on certain *Sphagnum* species from mire sites different in microrelief (ridge, carpet, hollow). *Sphagnum* moss samples for mite extraction were collected using a 10 × 10 cm frame to the depth of living moss plants (including capitula and the length of stems). Collected samples of moss substrates were placed in plastic zip bags and transported to the laboratory (Minor et al. 2016, Minor et al. 2019).

(g) Vertebrates. Along with studying other groups of organisms, visual observations of vertebrates and their traces were carried out (Philippov and Shabunov 2013). Whenever possible, animals and their traces were photographed, feathers or other fragments of animals or faeces were collected. Fishing was carried out with a float rod within legally-approved periods.

IV. Data collection: analysis of samples not identified in the field or verification of the identification data by the experts.

(a, b) Macrophytes and fungi. Herbarium materials of Tracheophyta, Bryophyta, Marchantiophyta and Ascomycota were transferred for processing to the Herbarium of the Mire Research Group of Papanin Institute for Biology of Inland Waters Russian Academy of Sciences (MIRE), while some doublets were transferred to VO, IBIW, PTZ and SYKO.

(c) Algae. Sedimented phytoplankton for qualitative and quantitative analysis was examined in a Nageotte counting chamber (0.01 cm^3^) using a ZeissAxiolab, NikonEclipse 80 i and XSZ-2101 (at 400x and 1000x magnification). Taxonomic identification was made to the closest possible low-range taxon.

(d) Protozoa. In the laboratory, heterotrophic flagellates and centrohelid heliozoans samples were enriched with a suspension of *Pseudomonasfluorescens* Migula bacteria at the ratio of 0.15 ml of suspension per 5 ml of sample and placed in Petri dishes. Samples were kept at 22°C in the dark and observed for 10 days to reveal the cryptic species diversity according to the accepted methodology (Vørs 1992). For observations, an AxioScope A1 light microscope (Carl Zeiss, Germany) with DIC and phase contrast and water immersion objectives (total magnification 1120x) was used. Video recording was made by an AVT HORN MC1009/S analogue video camera. Electron microscope preparations were carried out according to the approved method (Moestrup and Thomsen 1980) and observed in a JEM-1011 transmission electron microscope (Jeol, Japan). Testate amoebae samples were analysed immediately after transportation to the laboratory. NU-2E and Peraval-Interphako with water and oil immersion and an MBI-3 light microscope with a KF-5 phase-contrast installation in transmitted light were used. Analysis of heterotrophic flagellates, centrohelid heliozoans and testate amoebae abundance in the samples was not performed.

(e) Aquatic invertebrates. All specimens of zooplankton and zoobenthos were identified with an MBS-10 stereoscopic microscope and a Mikmed-6 microscope (LOMO, Russia). Aquatic insects were identified using Micromed MC-5-ZOOM LED and Leica M165C stereoscopic microscopes. These materials are deposited in the Papanin Institute for Biology of Inland Waters Russian Academy of Sciences (IBIW RAS): “Collection of autotrophic and heterotrophic organisms of mire ecosystems, IBIW RAS” and the entomologic collection.

(f) Terrestrial and soil invertebrates. On the day of sampling, sweep samples of terrestrial arthropods were primarily sorted by the main taxonomic groups (spiders, beetles, dipterans etc.). Separate samples were then fixed in ethanol. Detailed analysis, identification and counting were performed later by experts. Part of the collection was deposited in the Science Museum of the Institute of Biology of Komi Science Centre of the Ural Branch of the Russian Academy of Sciences. Mites from moss samples were extracted in modified Berlese funnels for five days. Adult Oribatida and Mesostigmata were identified to a species level and counted. Taxonomic identification of mites was carried out by the Acarology research group of Tyumen State University.

(g) Vertebrates. Found fragments of animals and their traces were collected and studied in the laboratory. Faunal lists were compiled.

Records list compilation. The dataset field names were chosen according to Darwin Core (Wieczorek et al. 2012). Georeferencing was made using a GPS navigator or Google maps. In all cases, the WGS-84 coordinate system is used.

## Geographic coverage

### Description

**Figure 1.**
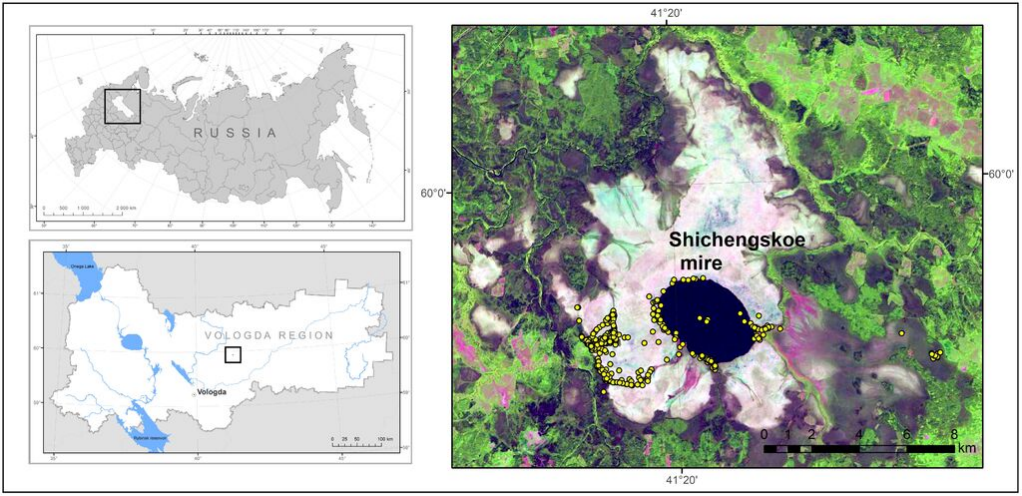
Location of the Vologda Region (top left), Shichengskoe mire (bottom left) and sampling sites (yellow circles on the right picture).

The study area is situated in the central part of the Vologda Region (59.8988 – 60.0590 N, 41.2327 – 41.5540 E), north-western Russia, the southern part of the middle taiga zone (Fig. 1). Shichengskoe wetland is a large mire area including the peat bog (Shichengskoe mire), intra-mire lakes (Shichengskoe Lake, Plakunovskoe Lake, Polyanok Lake) and rivers (Glukhaya Sondushka River, Sondushka River, Shichenga River), nameless mire streams and brooks, fen strips and lags, *Sphagnum* hollows, secondary hollow-pools and disturbed areas (Philippov 2017). Chemical characteristics of the intra-mire water objects were reported earlier (Philippov and Yurchenko 2020), as well as the data on microclimate differences between the mire sites (Philippov and Yurchenko 2019).

The study area is characterised by a temperate continental climate with long, cold, snowy winters, short springs with fluctuating temperatures, relatively short, moderately warm summers and long and rainy autumn. The average annual air temperature is + 1.5 to + 2.0ºC, the average monthly temperature in July is + 16 to + 17.0ºC, in January, -12.5 to -13.0ºC. The average annual precipitation ranges from 650 to 750 mm; during the active growing season, from 350 to 375 mm. The prevailing wind direction is southwest and south (Skupinova 2007).

The study area is confined to the Permian-Triassic plateau. The bedrock sedimentary rocks occur at a depth of 20–40 m and are represented by limestones and clays with lenses of sandy loam of the Tatar stage of the Permian system. The main features of the relief of the region are determined by glacial accumulation in the terminal moraines formed during the Moscow glaciation. Shichengskoe wetland was formed mainly by the limnogenic process on the south-eastern spurs of the Kharovsk ridge in a vast lacustrine-glacial basin. The ancient lake basin is orientated from northwest to southeast and reaches 20–25 km across. The bottom of the basin is a typical lacustrine-glacial plain with absolute heights of 130–150 m above sea level (Savinov and Romanova 1970). At the beginning of the Holocene, the post-glacial lake was significantly drained by rivers and streams, overgrowth and peat accumulation followed and a peat bog began to form in its place. Currently, a large mire with a residual lake fill up the Shichengskiy ancient lake basin.

The main aquifers are lacustrine, lacustrine-glacial and fluvioglacial intermoraine Quaternary sediments confined to sands, less often to interlayers of sands in sandy loams and clays. The area is provided with low-mineralised groundwater (Savinov and Filenko 1970). The surface waters of the Shichengskoe wetland belong to the regional drainage basin of Kubenskoe Lake, the global drainage basin of the Arctic Ocean (White Sea).

The soil-forming rocks in the area are moraines, enriched with boulders, sometimes carbonate material, less often fluvioglacial and binomial deposits being the parent rocks in the study area (Komissarov 1987). Directly on the territory of the Shichengskoe mire, soils are predominantly hydromorphic and semi-hydromorphic and peat soils prevail over the occupied area.

According to geobotanical zoning (Abramova and Kozlova 1970), the Shichengskoe wetland is located in the southern part of the Verknevazhsko-Kuloiskiy geobotanical district of haircap-moss and berry-grass spruce forests, pine and birch forests, transitional mires and raised bogs. In the region, almost half of the forest formations grow on soils of varying degrees of waterlogging due to insufficient drainage of the prevailing moraine and lacustrine-glacial plains composed of loams. About 80% of the forested area is occupied by pine forests with low quality of locality and represented mainly by swamp forest coenoses. About 10% of the forested area is occupied by spruce forests, of which paludified types prevail. Small-leaved forests are mainly represented by birch forests, which formed mainly at clearings (Bobrovskiy et al. 1993).

According to the classification proposed by T.K. Yurkovskaya (Sirin et al. 2017), Shichengskoe mire belongs to Pechora-Onezhskii raised bog type of the North-Eastern European *Sphagnum* raised bogs group of *Sphagnum* mires. Currently, the main part of the mire is at the oligotrophic stage of development; however, there are areas of eutrophic and mesotrophic types of water-mineral nutrition. The mire is located on the territory of the Shichengsko-Kuloiskiy mire district (Abramova 1965), which is paludified by more than 19% (Filonenko and Philippov 2013) and characterised by the predominance of forested mesotrophic and oligotrophic mires of lacustrine origin.

Since 1987, about 90% of the Shichengskoe wetland has belonged to the regional Shichengskiy Landscape Reserve. This Reserve is the largest landscape reserve in the Vologda Region (136.1 km^2^).

### Coordinates

59.923 and 59.965 Latitude; 41.259 and 41.531 Longitude.

## Taxonomic coverage

### Description

This dataset provides current data on vascular plants, cryptogams, microalgae and bacteria, protozoans, terrestrial, soil and aquatic invertebrates, as well as terrestrial and aquatic vertebrates in the Shichengskoe mire. The list consists of Animalia (5 phyla, 13 classes, 51 orders, 225 families), Bacteria (2 phyla, 2 classes, 5 orders, 9 families), Chromista (7 phyla, 7 classes, 23 orders, 41 families), Fungi (2 phyla, 2 classes, 9 orders, 22 families), Plantae (6 phyla, 13 classes, 52 orders, 105 families) and Protozoa (6 phyla, 9 classes, 13 orders, 27 families) species. Overall, the dataset comprises 1358 taxa, including 1250 lower-rank taxa (species, subspecies, varieties, forms) and 108 taxa identified to the genus level.

### Taxa included

**Table:** 

Rank	Scientific Name	
kingdom	Animalia	
kingdom	Bacteria	
kingdom	Chromista	
kingdom	Fungi	
kingdom	Plantae	
kingdom	Protozoa	

## Traits coverage

### Data coverage of traits

PLEASE FILL IN TRAIT INFORMATION HERE

## Temporal coverage

### Notes

1972, 1986, 2000 to 2021

## Usage licence

### Usage licence

Other

### IP rights notes

This work is licensed under a Creative Commons Attribution (CC-BY) 4.0 Licence.

## Data resources

### Data package title

Data on biodiversity of a boreal mire and its hydrographic network (Shichengskoe mire, North-Western Russia)

### Resource link

https://www.gbif.org/dataset/04209a70-813b-421a-a250-e893b8836cdc

### Alternative identifiers

http://gbif.ru:8080/ipt/resource?r=shichengskoe

### Number of data sets

1

### Data set 1.

#### Data set name

Data on biodiversity of a boreal mire and its hydrographic network (Shichengskoe mire, North-Western Russia)

#### Data format

Darwin Core

#### Number of columns

40

#### Character set

Occurrence dataset

#### Download URL

https://www.gbif.org/dataset/04209a70-813b-421a-a250-e893b8836cdc

#### Data format version

1.3

#### Description

This dataset provides current data on the biodiversity of Shichengskoe mire (Vologda Region, north-western Russia), including various mire sites and intra-mire water bodies. The data contain materials on the diversity of Animalia (2886 occurrences), Bacteria (22), Chromista (256), Fungi (111), Plantae (2463) and Protozoa (131). A total of 5869 occurrences (1250 lower-rank taxa and 108 taxa identified to the genus level) are included in the list.

**Data set 1.** 

Column label	Column description
occurrenceID	An identifier for the record, unique within this dataset. An abbreviation in the identifier' number (MiReGr_Shich_xxxxx).
basisOfRecord	The specific nature of the data record in standard label of one of the Darwin Core. A constant ("HumanObservation").
scientificName	The full scientific name, with authorship and date information, if known.
eventDate	The date or interval during which an event occurred. For occurrences, this is the date when the event was recorded. A variable.
taxonRank	The taxonomic rank.
kingdom	The full scientific name of the kingdom in which the taxon is classified.
phylum	The full scientific name of the phylum or division in which the taxon is classified.
class	The full scientific name of the class in which the taxon is classified.
order	The full scientific name of the order in which the taxon is classified.
family	The full scientific name of the family in which the taxon is classified.
genus	The full scientific name of the genus in which the taxon is classified.
habitat	A category or description of the habitat in which the Event occurred, in Russian. A variable.
decimalLatitude	The geographic latitude in decimal degrees of the geographic centre of the data sampling place.
decimalLongitude	The geographic longitude in decimal degrees of the geographic centre of the data sampling place.
geodeticDatum	The ellipsoid, geodetic datum or spatial reference system (SRS) upon which the geographic coordinates given in decimalLatitude and decimalLongitude are based. A constant ("WGS84").
coordinateUncertaintyInMetres	The maximum uncertainty distance in metres.
coordinatePrecision	A decimal representation of the precision of the coordinates given in the decimalLatitude and decimalLongitude. A constant ("0.0001").
countryCode	The standard code for the Russian Federation according to ISO 3166-1-alpha-2 (RU).
country	Country name (Russian Federation).
stateProvince	Region (‘oblast’) name. The first-level administrative division. A constant ("Vologda Region").
county	District (‘rayon’) name. The second-level administrative division. A constant ("Syamzhensky district").
locality	The specific description of the place. This term may contain information modified from the original to correct perceived errors or standardise the description. A variable (eight options: “Glukhaya Sondushka river”, “Plakunovskoe lake”, “Polyanok lake”, Shichenga river”, Shichengskoe lake”, “Shichengskoe mire”, Shichengskoe mire and lake”, “Sondushka river”).
individualCount	The number of individuals represented present at the time of the Occurrence.
sex	The sex (gender) of the taxon. A variable (male or female).
lifeStage	Period of lifespan development. A variable.
organismQuantity	Number or enumeration value for the quantity of organisms.
organismQuantityType	The type of quantification system used for the quantity of organisms. A variable (two options: "Braun-Blanquet scale", "percent cover").
sampleSizeValue	A numeric value for a measurement of the area.
sampleSizeUnit	The unit of measurement of the area. A constant ("m^2^").
year	The four-digit number of year in which the Event occurred, according to the Common Era Calendar.
month	The integer month in which the Event occurred.
day	The integer day of the month on which the Event occurred.
recordedBy	List of persons who collected field data.
identifiedBy	A person who assigned the Taxon to the subject.
dateIdentified	The date when the taxonomic identification happened.
associatedReferences	List of literature references associated with the occurrences.
language	A language of the resource (en | ru).
acceptedNameUsage	The full name, with authorship and date information, if known, of accepted taxon.
taxonomicStatus	The taxonomic status of a taxon. A variable (accepted or synonym).
taxonRemarks	Remarks regarding taxa.

## Additional information

The studied biotopes of the Shichengskoe mire were placed in the following groups:

(1) Mire expanse lake with its coastal area; this group combines Shichengskoe Lake, a 10.2 km^2^ flow-through shallow primary lake centrally situated in a mire expanse and the Lake’s paludified coastal area formed mainly by raised bog sites.

(2) Non-central mire lakes with coastal areas; this group includes two small lakes, Polyanok Lake and Plakunovskoe Lake, about 0.04 km^2^ each, non-flow-through 6-7 m deep primary lakes located closer to the edge of the Shichengskoe mire and lakes’ paludified coastal areas formed mainly by rich fen mire sites.

(3) Floating mats; this group includes peat-forming vegetation held together by roots and rhizomes and floating on water, developing in lakes and mire rivers.

(4) Mire rivers with banks; this group includes three small, 5 to 50 km long, rivers with river banks: Sondushka River and Glukhaya Sondushka River, draining into Shichengskoe Lake and Shichenga River, the outlet of Shichengskoe Lake.

(5) Mire streams with valleys; this group includes small watercourses with the weak flow, their paludified banks and weakly pronounced forested eutrophic valleys.

(6) Fen strip sites; this group includes structural elements of fen strips, specific water objects forming solely in mires, narrow mire areas receiving an inflow of water from the surrounding mire, almost without trees, with meso- or meso-oligotrophic with grass and grass-moss communities. In the Shichengskoe mire, these flow-through fen strips begin at the intra-mire islands.

(7) Rich fen sites; this group includes rich fens, peatlands receiving an inflow of water from the mineral soil, located closer to the mire’s edge, having groundwater outlets and eutrophic peat.

(8) Raised bog feature and its elements; this group includes ridges, hummocks, lawns, *Sphagnum* hollows and secondary hollow-pools, the structural elements of oligotrophic mire sites (that occupy the most significant area in the Shichengskoe mirе), underlain by oligotrophic peat and having a set of plant communities characteristic of the taiga zone. Often these structural elements in various combinations form patterns, for example, a ridge-hollow pattern.

(9) Margins and edges; this group includes margins of a mire massif and paludified edges of intra-mire mineral islands.

(10) Disturbed areas; this group includes burnt places and bonfires, fishing grounds, trails and roads in a mire.

(11) Other biotopes; this group includes biotopes that did not fall into any of the previous groups and the occurrences of migratory birds and some mammals that pass through the mire or use several biotopes.

Examples of these biotopes are given in figures (Figs 2, 3, 4, 5, 6, 7, 8, 9, 10, 11, 12, 13, 14).

The studied biotobe groups were investigated unevenly (Table 1). A strong correlation was found between the number of occurrences and the number of lower-rank taxa found in the groups of biotopes (Spearman Rank Correlation Coefficient 0.98, p < 0.05). The greatest number of occurrences came from the raised bog features and their elements and fen strip sites which accounted for 359 and 371 lower-rank taxa, respectively. Mire streams with their valleys and margins and edges were studied much less, but these biotopes also showed significant biodiversity.

Half of the total occurrences came from the intra-mire water bodies that comprised 59.6% of lower-rank taxa (Table 2). The most studied were fen strips and this biotope group showed the most significant biodiversity. It is worth noting that the second largest number of lower-rank taxa was found in Shichengskoe Lake, the mire expanse lake, the largest in the studied mire complex; it provided one-third of the lower-rank taxa, based on almost one-eighth of the number of occurrences.

Table 3 shows the distribution of species and lower-rank taxa recorded on the Shichengskoe mire by major taxonomic groups. This data allows us to conceive of the level of biodiversity exploration and prospects for further research.

During the studies, we found a significant amount of endangered species within the Shichengskoe wetland, five included in the Red Data Book of the Russian Federation (Danilov-Danilyan 2001, Bardunov and Novikov 2008): a stonewort *Charastrigosa*, a hawker dragonfly *Anaximperator*, a raptor *Pandionhaliaetus*, a wader *Numeniusarquata* and a songbird *Laniusexcurbitor*. Notably, the first two species have not been included in the Red Data Book of the Vologda Region because no confirmed findings had been known in the region at the time. In the Red Data Book of the Vologda Region, 57 species found in the Shichengskoe wetland are listed (Bolotova et al. 2010, Suslova et al. 2013, Anonymous 2015). According to the IUCN status, Critically Endangered (CR) – *Saxifragahirculus*; Endangered (EN): *Hammarbyapaludosa*; Vulnerable (VU): *Sphagnumlindbergii*, *Cygnuscygnus*, *Lagopuslagopus*, and *Limosalimosa*; Near Threatened (NT): *Droseraanglica*, *Utriculariaminor*, *Dactylorhizabaltica*, *Trichophorumalpinum* [as *Baeothryonalpinum* (L.) Egor.], Carexoederivar.oederi [as *C.serotina* Merat.], *Rhynchosporaalba*, *Grusgrus*, and *Numeniusphaeopus*; Least Concern (LC): *Ligulariasibirica*, *Petasitesfrigidus*, *Malaxismonophyllos*, *Carexpseudocyperus*, *Papiliomachaon*, and *Milvusmigrans*; Data Deficient (DD): *Oxycoccusmicrocarpus*, *Cladoniastygia* and *Ramalinadilacerata*. Amongst the 57 species listed in the Red Data Book of the Vologda Region, there are 35 rare species marked as biological control required: *Nymphaeacandida*, *Rumexhydrolapathum*, *Betulahumilis*, *Betulaintermedia*, *Monesesuniflora*, *Empetrumhermaphroditum*, *Salix×holosericea* [as *S.dasyclados* Wimm.], *Salixlapponum*, *Daphnemezereum*, *Rubusarcticus*, *Galiumtriflorum*, *Utriculariaintermedia*, *Dactylorhizafuchsii*, *Dactylorhizaincarnata*, *Dactylorhizarussowii*, *Epipactispalustris*, *Gymnadeniaconopsea*, *Platantherabifolia*, *Scolochloafestucacea*, *Hydrocharismorsus-ranae*, *Potamogetonberchtoldii*, *Potamogetonpraelongus*, *Sparganiumnatans*, *Typhaangustifolia*, *Sphagnumsubsecundum*, *Sphagnumwulfianum*, *Meesialongiseta*, *Scapaniapaludicola*, *Icmadophilaericetorum*, *Gammaruspulex*, *Coliaspalaeno*, *Bombusjonellus*, *Ardeacinerea* and *Lutralutra*. However, only 80% of the total number of rare and protected species was registered within the boundaries of the Shichengskiy Landscape Reserve.

## Figures and Tables

**Figure 2.**
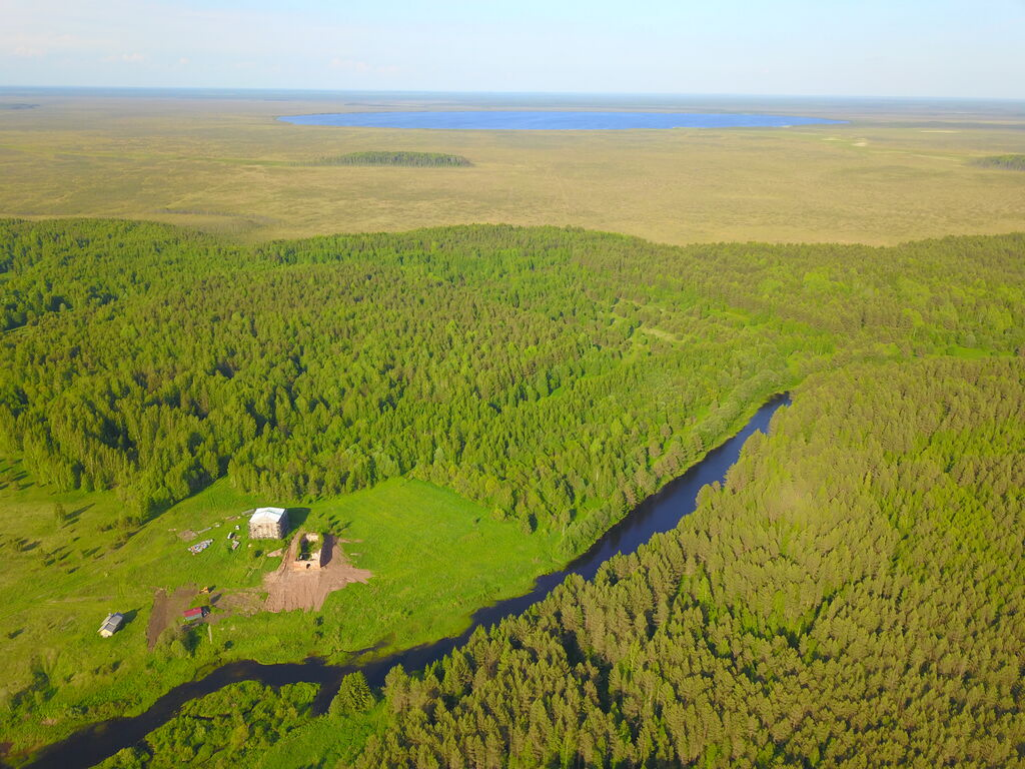
General view of Shichengskoe mire with its mire expanse lake (background) and adjoining mineral soil (foreground), Vologda Region, Russia. Photo by Dmitriy A. Philippov (2019).

**Figure 3.**
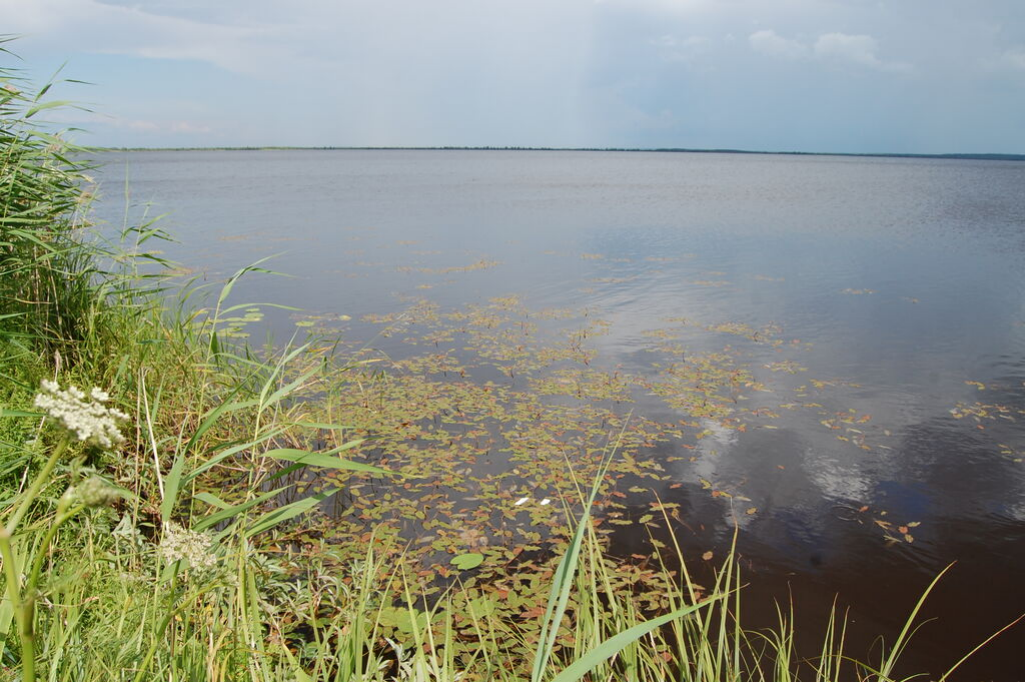
Shichengskoe Lake, a mire expanse lake (Vologda Region, Russia). Photo by Dmitriy A. Philippov (2014).

**Figure 4.**
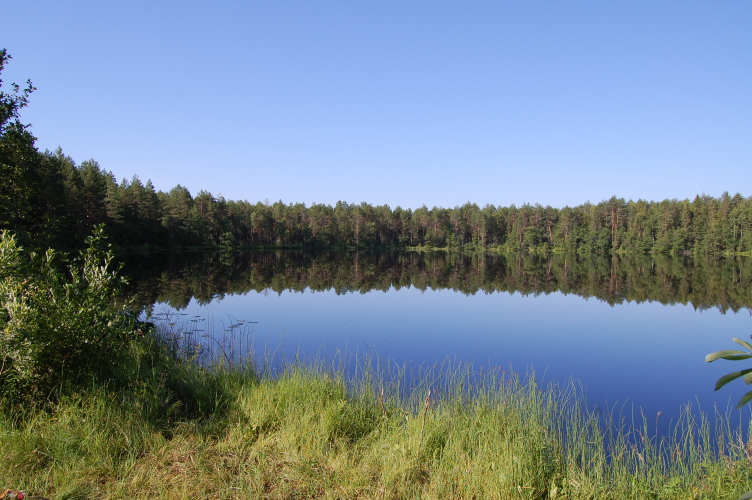
Polyanok Lake, a non-central mire lake (Vologda Region, Russia). Photo by Dmitriy A. Philippov (2014).

**Figure 5.**
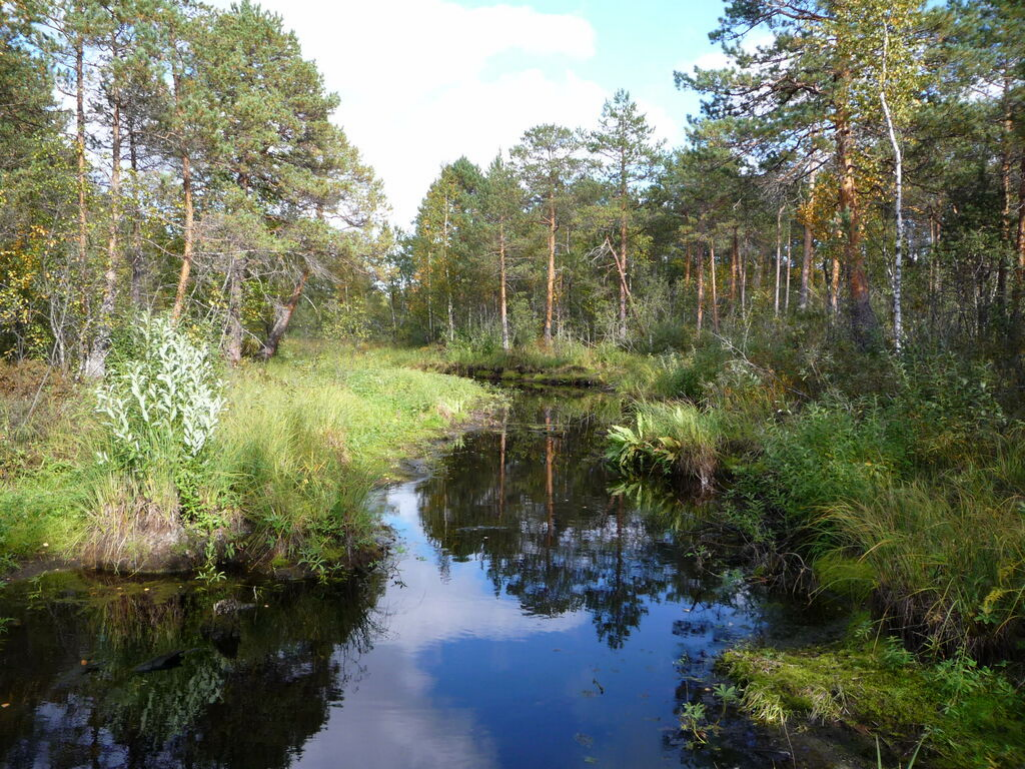
Glukhaya Sondushka River, a mire river (Vologda Region, Russia). Photo by Dmitriy A. Philippov (2010).

**Figure 6.**
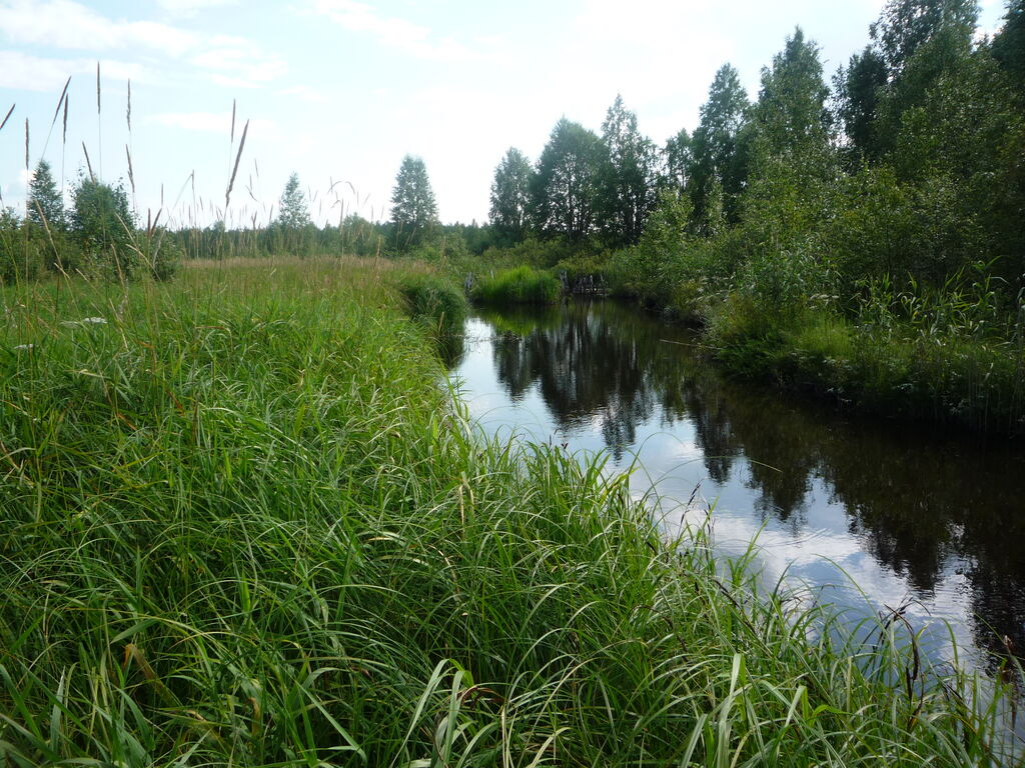
Shichenga River, a mire river (Vologda Region, Russia). Photo by Dmitriy A. Philippov (2011).

**Figure 7.**
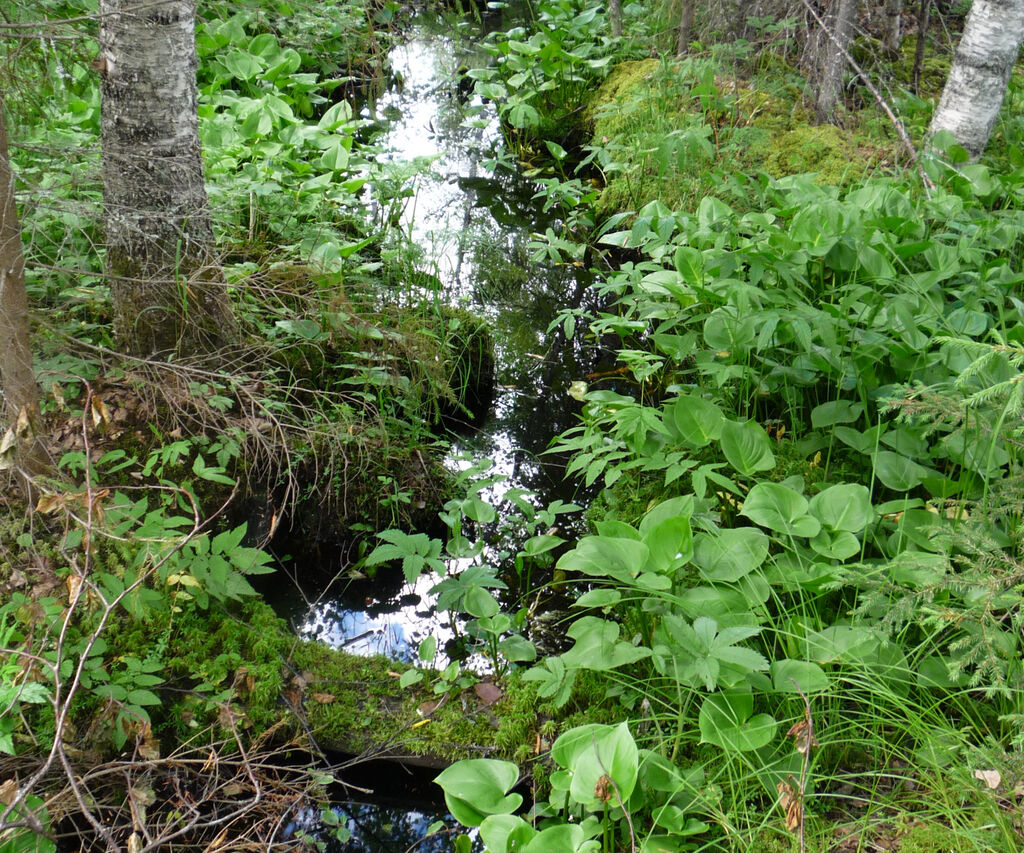
A mire stream on a mire margin, Shichengskoe mire (Vologda Region, Russia). Photo by Dmitriy A. Philippov (2012).

**Figure 8.**
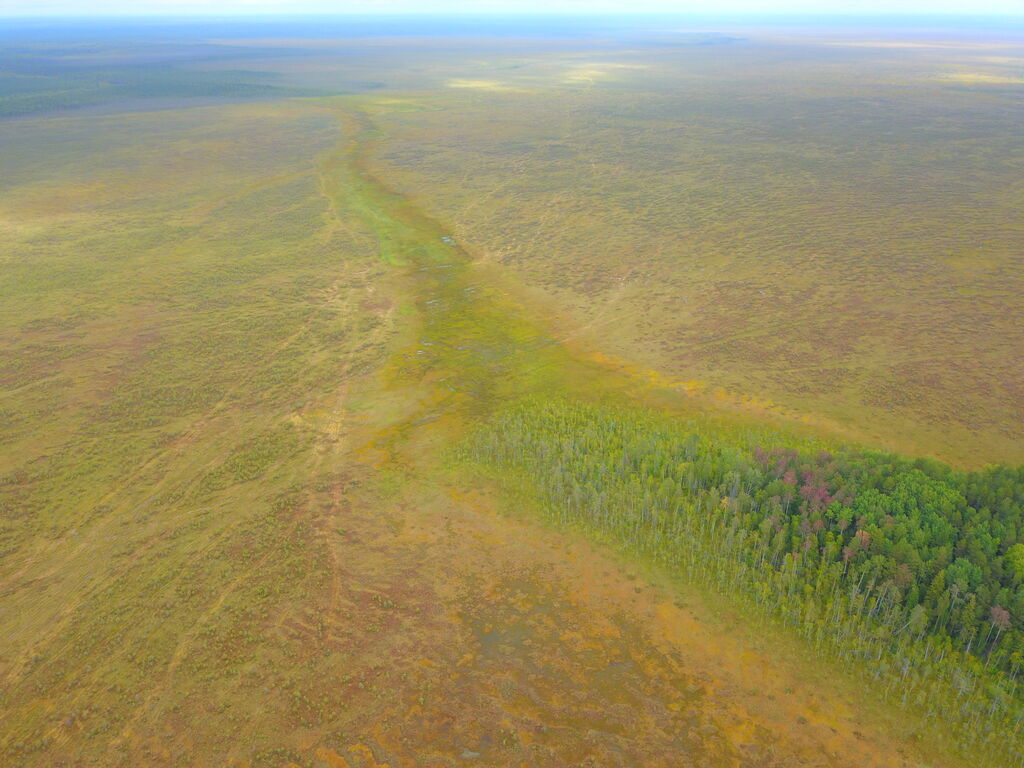
General view of a fen strip, Shichengskoe mire (Vologda Region, Russia). Photo by Dmitriy A. Philippov (2019).

**Figure 9.**
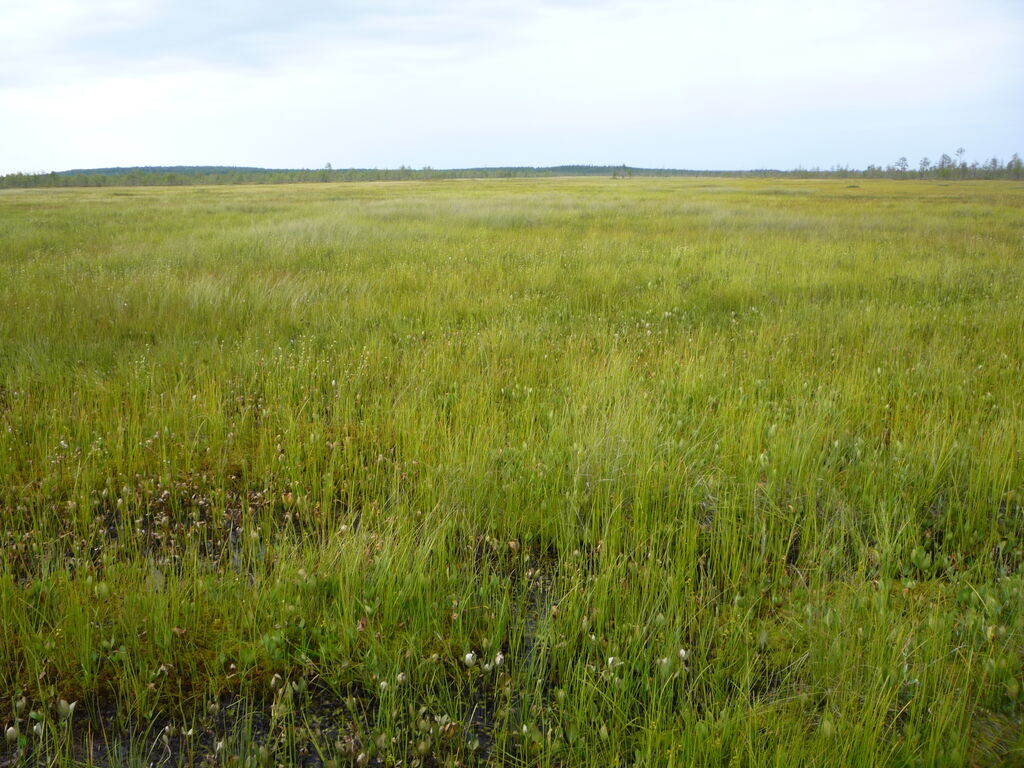
Herbaceous communities in a fen strip, Shichengskoe mire (Vologda Region, Russia). Photo by Dmitriy A. Philippov (2015).

**Figure 10.**
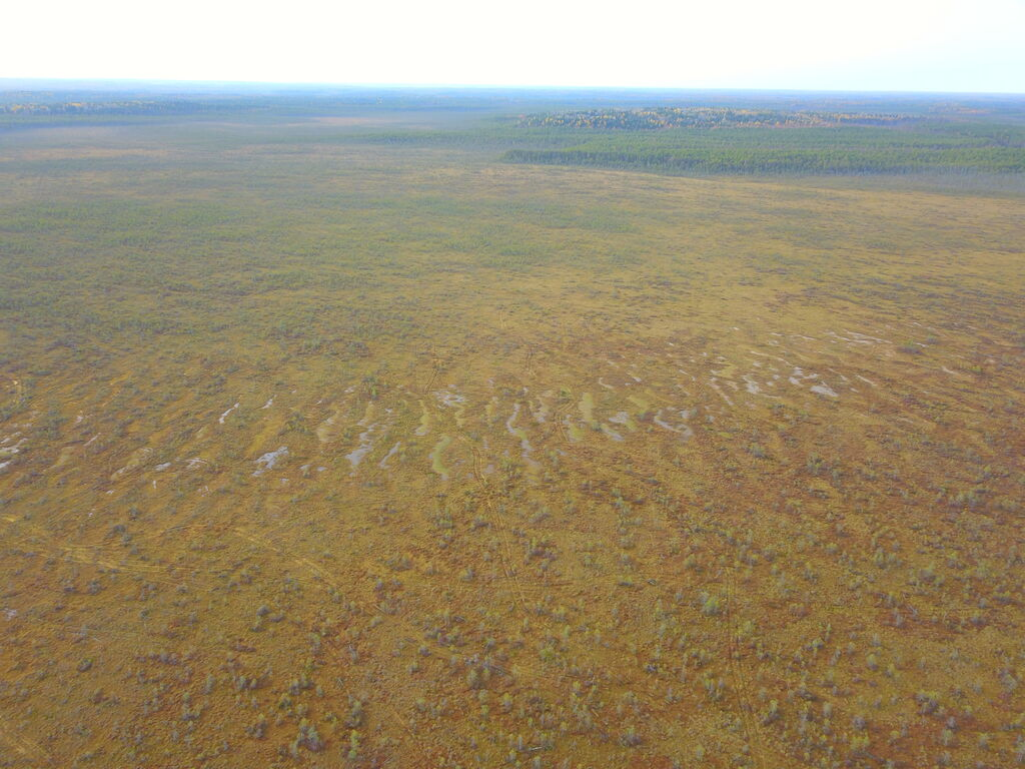
General view of a raised bog part including a ridge-hollow pattern, Shichengskoe mire (Vologda Region, Russia). Photo by Dmitriy A. Philippov (2020).

**Figure 11.**
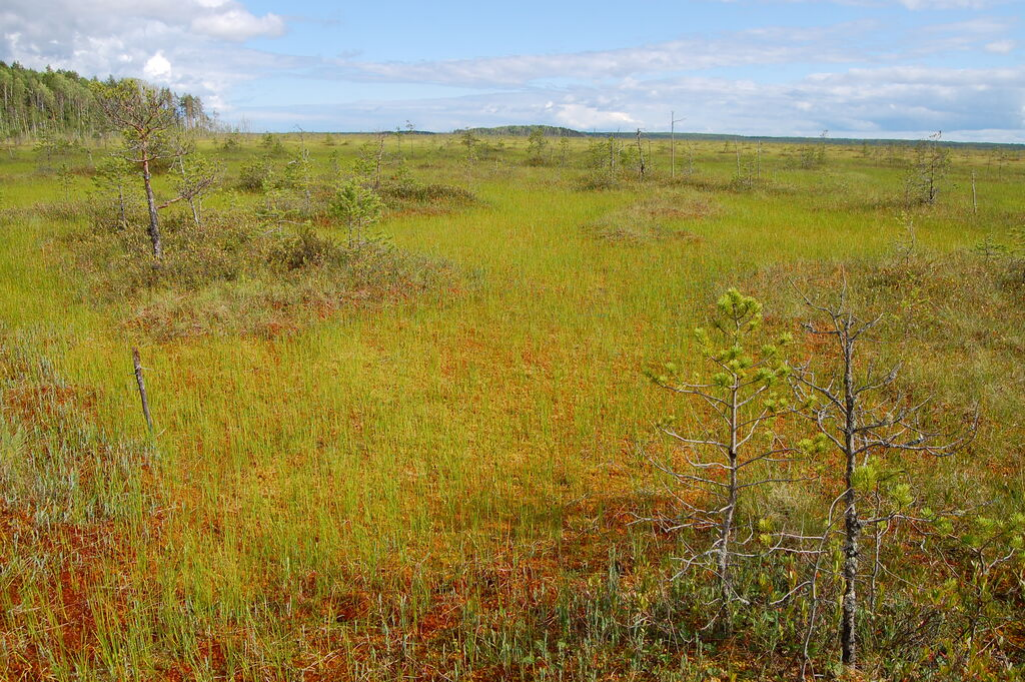
A ridge-hollow pattern in a raised bog part, Shichengskoe mire (Vologda Region, Russia). Photo by Dmitriy A. Philippov (2015).

**Figure 12.**
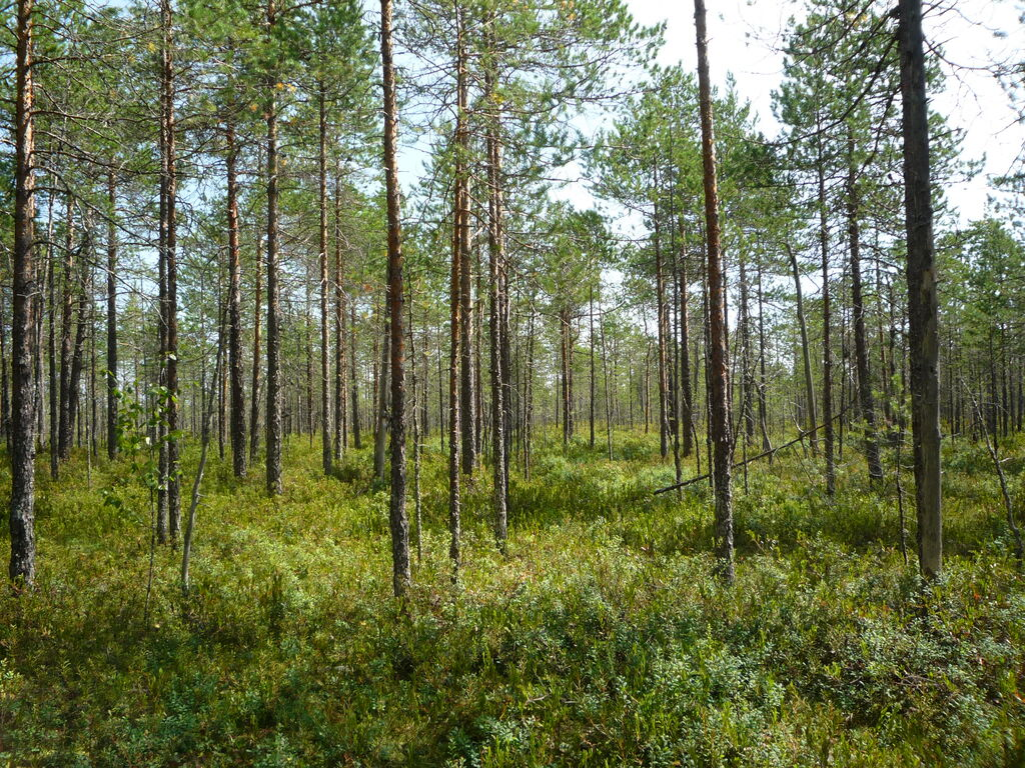
A paludified oligotrophic peat-moss pine forest on a mire margin, Shichengskoe mire (Vologda Region, Russia). Photo by Dmitriy A. Philippov (2015).

**Figure 13.**
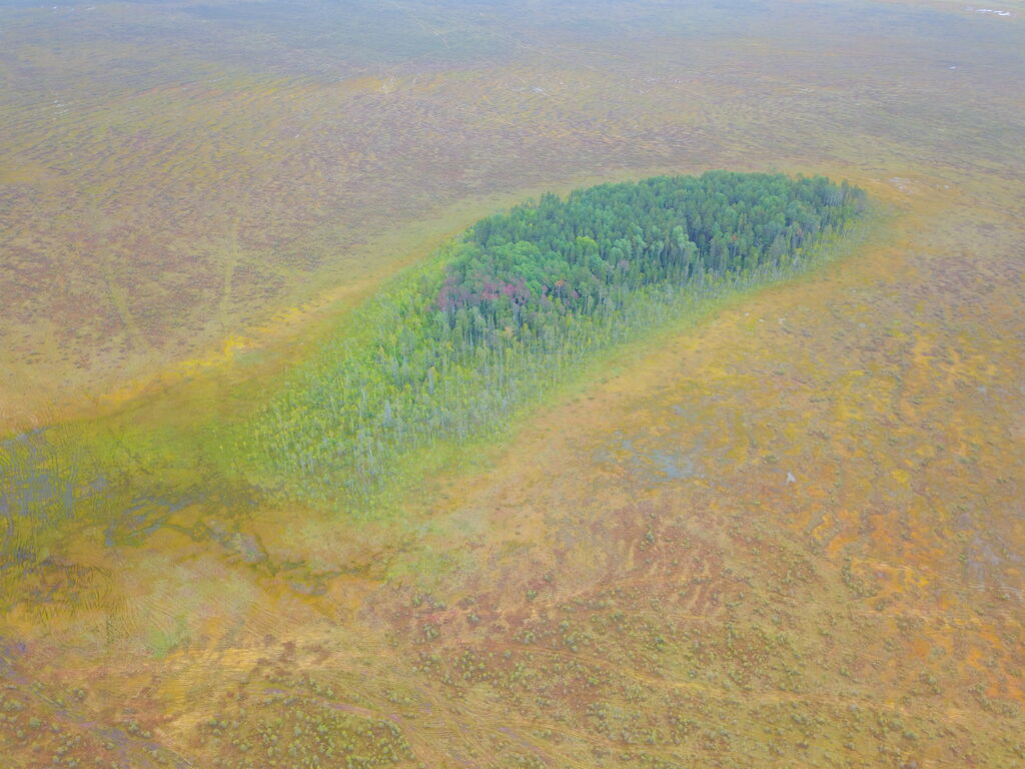
An intra-mire mineral island in Shichengskoe mire (Vologda Region, Russia). Photo by Dmitriy A. Philippov (2019).

**Figure 14.**
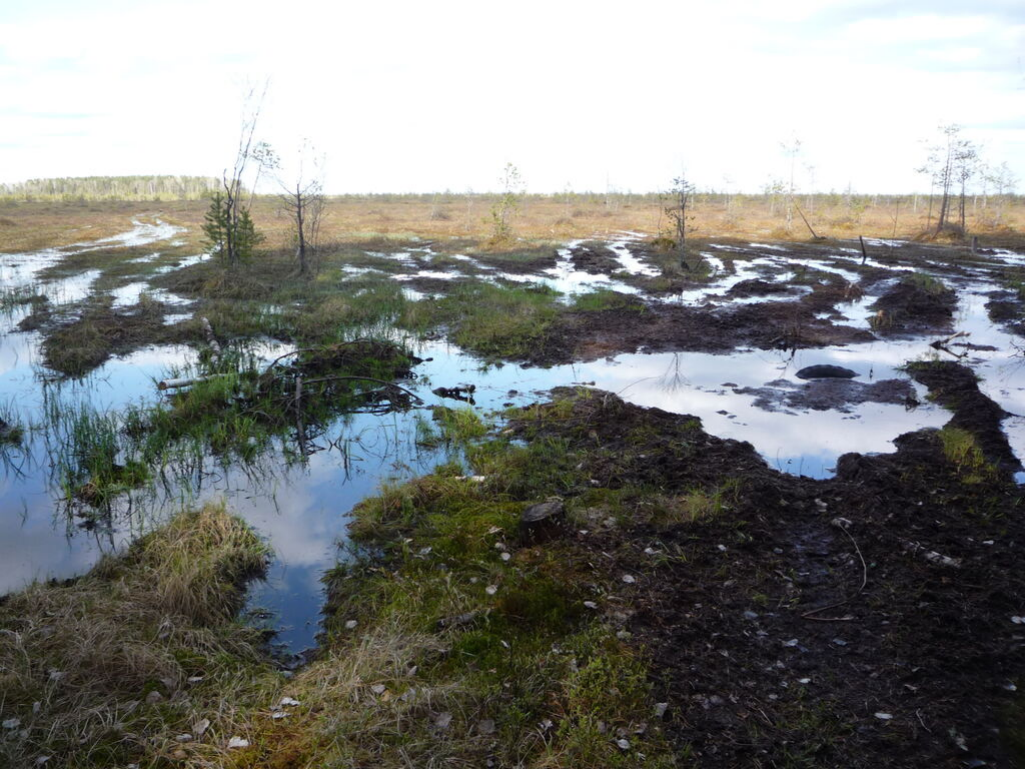
Swamp-mobile tracks, an example of a disturbed area in the Shichengskoe mire (Vologda Region, Russia). Photo by Dmitriy A. Philippov (2017).

**Table 1.** Number of lower-rank taxa (species, subspecies, varieties, forms) in groups of biotopes of Shichengskoe mire (Vologda Region, Russia).

Main mire parts	Number of occurrences	Number of lower-rank taxa						
		Total	Animalia	Bacteria	Chromista	Fungi	Plantae	Protozoa
Shichengskoe mire with its network	**5611**	**1250**	**586**	**10**	**118**	**50**	**423**	**63**
Mire expanse lake with its coastal area	468	287	109	4	60	2	98	14
Non-central mire lakes with coastal areas	151	127	67				60	
Floating mats	53	44					44	
Mire rivers with banks	105	61	6				55	
Mire streams with valleys	593	351	190	2	42		100	17
Fen strip sites	1232	371	236	3	36	2	74	20
Rich fen sites	437	213	48			7	158	
Raised bog features and their elements	1855	359	221	2	30	18	61	27
Margins and edges	623	314	94		2	36	166	16
Disturbed areas	74	57	1				56	
Other biotopes	20	15	12			2	1	

**Table 2.** Number of lower-rank taxa (species, subspecies, varieties, forms) in the mire water object of Shichengskoe mire (Vologda Region, Russia).

Water object	Number of occurrences	Number of lower-rank taxa						
		Total	Animalia	Bacteria	Chromista	Fungi	Plantae	Protozoa
Shichengskoe mire with its network	**5611**	**1250**	**586**	**10**	**118**	**50**	**423**	**63**
Mire waterbodies (total)	**2806**	**744**	**365**	**10**	**117**	**2**	**205**	**45**
Mire expanse lake	356	231	78	4	60		75	14
Non-central mire lakes	118	108	67				41	
Floating mats	53	44					44	
Mire rivers	78	48	4				44	
Mire streams	308	176	69	2	42		51	12
Fen strips	1232	371	236	3	36	2	74	20
*Sphagnum* hollows	568	132	77	2	15		27	11
Hollow-pools	93	75	24	1	24		21	5

**Table 3.** Numbers of lower-rank taxa (species, subspecies, varieties, forms) and species in higher-rank taxa (kingdom, phylum) registered in the Shichengskoe mire (Vologda Region, Russia)

**Kingdom, phylum**	**Number of lower-rank taxa**	**Number of species**
**Animalia**	**586**	**581**
Annelida	15	15
Arthropoda	441	436
Chordata	87	87
Mollusca	2	2
Rotifera	40	40
phylum not specified	1	1
**Bacteria**	**10**	**10**
Cyanobacteria	9	9
Proteobacteria	1	1
**Chromista**	**118**	**92**
Bigyra	1	1
Cercozoa	5	5
Cryptophyta	1	1
Foraminifera	1	1
Heliozoa	7	7
Myzozoa	3	3
Ochrophyta	100	74
**Fungi**	**50**	**46**
Ascomycota	38	34
Basidiomycota	12	12
**Plantae**	**423**	**402**
Bryophyta	65	65
Charophyta	46	41
Chlorophyta	21	21
Marchantiophyta	39	37
Tracheophyta	252	238
**Protozoa**	**63**	**61**
Amoebozoa	30	28
Choanozoa	4	4
Euglenozoa	13	13
Loukozoa	2	2
Sulcozoa	2	2
phylum not specified	12	12
Total:	**1250**	**1192**

## References

[B7512028] Abolin A. A., Afonina O. M., Andreeva E. N., Badmaeva N. K., Bakalin V. A., Belkina O. A., Borovichev E. A., Chemeris E. V., Cherdantseva V. Y., Cherednichenko O. V., Czernyadjeva I. V., Doroshina G. Y., Dulin M. V., Ibatullin A. A., Ignatov M. S., Ignatova E. A., Kokoshnikova Y. S., Kotseruba V. V., Konstantinova N. A., Malashkina E. V., Mamontov Y. S., Notov A. A., Opmanis A. G., Philippov D. A., Potemkin A. D., Reriha I. S., Schestakova A. A., Schilnikov D. S., Sofronova E. V., Susko U. A., Teleganova V. V., Tubanova D. Y. (2011). New records. Arctoa.

[B7565196] Abramova T. G., Zubkov A. I. (1965). North-West European part of the USSR, vol. 4.

[B7565209] Abramova T. G., Kozlova G. I., Davydov L. K. (1970). Natural zoning of the Vologda region for agricultural purposes.

[B7512520] Afonina O. M., Akatova T. V., Andrejeva E. N., Baisheva E. Z., Belkina O. A., Bezgodov A. G., Borovichev E. A., Boychuk M. A., Czernyadjeva I. V., Doroshina G. Y., Dulin M. V., Fedosov V. E., Ignatov M. S., Ignatova E. A., Konstantinova N. A., Krivobokov L. V., Kučera J., Kushnevskaja E. V., Maksimov A. I., Maksimova T. A., Mamontov Y. S., Notov A. A., Philippov D. A., Potemkin A. D., Ryazanova D. T., Savchenko A. N., Schilnikov D. S., Sofronova E. V., Tubanova D. Y., Urbanavichus G. P., Urbanavichene I. N., Volkova E. M., Zheleznova G. V., Zolotov V. I. (2010). New records. Arctoa.

[B7517159] (2015). Resolution of the government of Vologda Region from 24.02.2015 №125 «On approval of list of rare and endangered species (intraspecific taxa) plants and fungi, which feature in the Red Data Book of Vologda Region». https://vologda-oblast.ru/dokumenty/393552/.

[B7517179] Bardunov L. V., Novikov V. S. (2008). Красная книга Российской Федерации (растения и грибы).

[B7511894] Bengtsson F., Rydin H., Baltzer J. L., Bragazza L., Bu Z. J., Caporn S. J.M., Dorrepaal E., Flatberg K. I., Galanina O., Gałka M., Ganeva A., Goia I., Goncharova N., Hájek M., Haraguchi A., Harris L. I., Humphreys E., Jiroušek M., Kajukało K., Karofeld E., Koronatova N. G., Kosykh N. P., Laine A. M., Lamentowicz M, Lapshina E., Limpens J., Linkosalmi M., Ma J. Z., Mauritz M., Mitchell E. A.D., Munir T. M., Natali S. M., Natcheva R., Payne R. J., Philippov D. A., Rice S. K., Robinson S., Robroek B. J.M., Rochefort L., Singer D., Stenøien H. K., Tuittila E. S., Vellak K., Waddington J. M., Granath G. (2021). Environmental drivers of *Sphagnum* growth in peatlands across the Holarctic region. Journal of Ecology.

[B7511650] Bobroff Y. A., Pozdeeva L. M., Philippov D. A. (2017). Variation in biomorphological structure of mire flora during the evolution of its surface hydrographic network. Transactions of Papanin Institute for Biology of Inland Waters RAS.

[B7511957] Bobrovskiy R. V., Vorobyev G. A., Komissarov V. V., Ukhanov V. P., Shevelev N. N., Vorobyev G. A. (1993). Specially protected natural areas, plants and animals of Vologda Region.

[B7517131] Bolotova N. L., Ivanter E. V., Krivokhatsky V. A. (2010). Красная книга Вологодской области. Т. 3. Животные.

[B7511569] Czhobadze A. B., Philippov D. A. (2015). New location of protected species of lichens in the Vologda Region. Phytodiversity of Eastern Europe.

[B7517167] Danilov-Danilyan V. I. (2001). Красная книга Российской Федерации (Животные).

[B7517040] Dedysh S. N., Beletsky A. V., Ivanova A. A., Kulichevskaya I. S., Suzina N. E., Philippov D. A., Rakitin A. L., Mardanov A. V., Ravin N. V. (2021). Wide distribution of *Phycisphaera*‐like planctomycetes from WD2101 soil group in peatlands and genome analysis of the first cultivated representative. Environmental Microbiology.

[B7517121] Dulin M. V., Philippov D. A. (2010). Additions to the liverworts flora of the Vologda Region. Herald of Tver State University. Series: Biology and Ecology.

[B7511884] Evgrafova I. (2004). Environmental certification of Syamzha District lakes. Izvestiya of the Vologda Society for the Study of the Northern Kray.

[B7565186] Filonenko I. V., Philippov D. A. (2013). Estimation of the area of mires in Vologda Region. Proceedings of Instorf.

[B7517447] Golub V. B., Tsurikov M. N., Prokin A. A. (2021). Коллекции насекомых: сбор, обработка и хранение материала.

[B7511971] Ivanova A. A., Beletsky A. V., Rakitin A. L., Kadnikov V. V., Philippov D. A., Mardanov A. V., Ravin N. V., Dedysh S. N. (2020). Closely located but totally distinct: highly contrasting prokaryotic diversity patterns in raised bogs and eutrophic fens. Microorganisms.

[B7511985] Ivicheva K. N., Philippov D. A. (2013). On macrozoophytes in *Fontinalisantipyretica* communities in ponds and streams of the Vologda region. Yaroslavl Pedagogical Bulletin.

[B7511995] Ivicheva K. N., Philippov D. A. (2015). *Anaximperator* (Insecta, Odonata) in the Vologda Region. International Journal of Applied and Fundamental Research.

[B7512018] Ivicheva K. N., Philippov D. A. (2017). Aquatic macroinvertebrates of raised bogs in the central part of the Vologda Region, Russia. Transactions of the Karelian Research Centre of the Russian Academy of Sciences.

[B7513230] Kapustin D. A., Philippov D. A., Gusev E. S. (2016). Four new chrysophycean stomatocysts with true complex collar from the Shichengskoe raised bog in Central Russia. Phytotaxa.

[B7511864] Kapustin D. A., Philippov D. A., Sokolova I. V., Gusev E. S. (2016). *Petalomonassphagnophila* (Euglenophyta, Petalomonadales), a new euglenophyte species for Russia. Novosti Systematiki Nizshikh Rasteniy.

[B7565246] Komissarov V. V. (1987). Почвы Вологодской области, их рациональное использование и охрана.

[B7511874] Lobunicheva E. V., Philippov D. A. (2017). Zooplankton of intramire primary lakes of the Shichengskoe mire (Vologda Region, Russia). Transactions of Papanin Institute for Biology of Inland Waters RAS.

[B7511841] Minor M. A., Ermilov S. G., Philippov D. A., Prokin A. A. (2016). Relative importance of local habitat complexity and regional factors for assemblages of oribatid mites (Acari: Oribatida) in *Sphagnum* peat bogs. Experimental and Applied Acarology.

[B7511831] Minor M. A., Ermilov S. G., Philippov D. A. (2019). Hydrology-driven environmental variability determines abiotic characteristics and Oribatida diversity patterns in a *Sphagnum* peatland system. Experimental and Applied Acarology.

[B7513217] Moestrup Ø., Thomsen H. A., Stein-Taylor J. R., Gantt E. (1980). Handbook of Phycological Methods: Developmental and Cytological Methods.

[B7512511] Pestov S. V., Philippov D. A. (2016). Diptera of the Shichengskoe mire (Vologda Region, Russia).

[B7511579] Pestov S. V., Philippov D. A. (2021). Structure of the plant-inhabiting insect fauna in a middle-taiga mire (Vologda Region, Russia). Theoretical and Applied Ecology.

[B7565288] Philippov D. A. (2010). Растительный покров, почвы и животный мир Вологодской области (ретроспективный библиографический указатель).

[B7511821] Philippov D. A., Shabunov A. A. (2013). On avifauna of the Shichengskoe bog (Vologda Oblast). Russian Ornitological Journal.

[B7517077] Philippov D. A. (2014). Hydrochemical characteristics of mire water tracks (by the example of Shichengskoe raised bog, Vologda Region). Water: Chemistry and Ecology.

[B7511731] Philippov D. A., Shabunov A. A. (2014). The common crane *Grusgrus* in the Vologda Oblast. Russian Ornithological Journal.

[B7511761] Philippov D. A. (2015). Flora of wetland "Shichengskoe" (Vologda Region, Russia). Phytodiversity of Eastern Europe.

[B7511771] Philippov D. A. (2015). On the records of some rare insects in the Vologda Region. International Journal of Applied and Fundamental Research.

[B7511781] Philippov D. A. (2015). *Oxycoccusmicrocarpus* (Ericaceae) in the Vologda Region. Phytodiversity of Eastern Europe.

[B7511791] Philippov D. A. (2015). *Papiliomachaon* Linnaeus, 1758 (Insecta, Lepidoptera, Papilionidae) in the Vologda Region. International Journal of Applied and Fundamental Research.

[B7511801] Philippov D. A., Boychuk M. A. (2015). Mosses of the Shichengskiy Landscape Reserve (Vologda Region). Vestnik of Northern (Arctic) Federal University. Seria Natural Sciences.

[B7511811] Philippov D. A., Dulin M. V. (2015). Liverworts of the Shichengskiy Landscape Reserve (Vologda region). Bulletin of Bryansk Department of Russian Botanical Society.

[B7511751] Philippov D. A. (2016). New data about rare birds of the Vologda Oblast. Russian Ornithological Journal.

[B7512598] Philippov D. A. (2017). Specific features of structural organization of hydrobiocenoses in different-type of mire water bodies and water courses. Transactions of Papanin Institute for Biology of Inland Waters RAS.

[B7511741] Philippov D. A., Leonov M. M. (2017). First data on testate amoebas (Testacea) in mires of Vologda Region, Russia. Transactions of Papanin Institute for Biology of Inland Waters RAS.

[B7513196] Philippov D. A., Prokin A. A., Przhiboro A. A. (2017). Методы и методики гидробиологического исследования болот: учебное пособие.

[B7512982] Philippov D. A., Yurchenko V. V. (2019). Data on air temperature, relative humidity and dew point in a boreal *Sphagnum* bog and an upland site (Shichengskoe mire system, North-Western Russia). Data in Brief.

[B7512968] Philippov D. A., Yurchenko V. V. (2020). Data on chemical characteristics of waters in two boreal *Sphagnum* mires (North-Western Russia). Data in Brief.

[B7515095] Philippov D. A., Ermilov S. G., Zaytseva V. L., Pestov S. V., Kuzmin E. A., Shabalina J. N., Sazhnev A. S., Ivicheva K. N., Sterlyagova I. N., Leonov M. M., Boychuk M. A., Czhobadze A. B., Prokina K. I., Dulin M. V., Joharchi O., Shabunov A. A., Shiryaeva O. S., Levashov A. N., Komarova A. S., Yurchenko V. V. (2021). Data on biodiversity of a boreal mire and its hydrographic network (Shichengskoe mire, North-Western Russia). https://www.gbif.org/dataset/04209a70-813b-421a-a250-e893b8836cdc.

[B7512491] Prokin A. A., Petrov P. N., Sazhnev A. S., Stolbov V. A., Philippov D. A. (2016). New records of water beetles (Coleoptera: Dytiscidae, Gyrinidae, Hydrophilidae) from Vologda and Tyumen Oblasts, Russia.

[B7517097] Prokina K. I., Philippov D. A., Mylnikov A. P. (2016). On heterotrophic flagellates of raised bogs *Sphagnum* hollows in the North of European Russia.

[B7511599] Prokina K. I., Zagumyonnyi D. G., Philippov D. A. (2017). Centrohelids in the mires of Northern Russia. Protistology.

[B7511589] Prokina K. I., Philippov D. A. (2018). Heterotrophic flagellates in the primary lakes and hollow-pools of mires in the European North of Russia. Protistology.

[B7517151] Prokina K. I. (2020). Видовое разнообразие и морфология гетеротрофных жгутиконосцев и центрохелидных солнечников разнотипных водных экосистем: Диссертация кандидата биологических наук.

[B7565275] Savinov Y. A., Filenko R. A., Davydov L. K. (1970). Natural zoning of the Vologda region for agricultural purposes.

[B7565262] Savinov Y. A., Romanova V. P., Davydov L. K. (1970). Natural zoning of the Vologda region for agricultural purposes.

[B7511640] Sazhnev A. S., Philippov D. A. (2017). On aquatic and amphibiotic beetles (Insecta: Coleoptera) of mire water bodies of Vologda Region, Russia. Transactions of Papanin Institute for Biology of Inland Waters RAS.

[B7511701] Sazhnev A. S., Zabaluev I. A., Philippov D. A. (2017). Rare and new beetles (Coleoptera) for the fauna of the Vologda Province. Eversmannia.

[B7511630] Sazhnev A. S., Pestov S. V., Philippov D. A. (2019). Review of leaf-beetles (Coleoptera: Chrysomelidae) in mires of Vologda Region, Russia. Proceeding of the Mordovia State Nature Reserve.

[B7511609] Sazhnev A. S., Ivicheva K. N., Komarova A. S., Philippov D. A. (2019). A review of aquatic, semi-aquatic and amphibiotic beetles (Insecta: Coleoptera) of Vologodskaya Oblast, Russia. Evraziatskii Entomologicheskii Zhurnal.

[B7511619] Sazhnev A. S., Komarova A. S., Philippov D. A. (2020). New records of aquatic beetles (Insecta: Coleoptera) for the fauna of Vologodskaya Oblast, Russia. Evraziatskii Entomologicheskii Zhurnal.

[B7511691] Shabunov A. A., Philippov D. A. (2018). The grey heron *Ardeacinerea* in the Vologda Oblast. Russian Ornithological Journal.

[B7511711] Shabunov A. A., Komarova A. S., Philippov D. A. (2019). New records of rare birds in the Vologda Oblast (according to observations of 2019). Russian Ornithological Journal.

[B7517106] Shevchenko V. P., Philippov D. A., Politova N. V., Starodymova D. P., Aliev R. A., Pokrovsky O. S., Pokrovsky O., Volkova I., Kosykh N., Shevchenko V. (2018). Mosses: Ecology, life cycle and significance.

[B7565231] Sirin A., Minayeva T., Yurkovskaya T., Kuznetsov O., Smagin V., Fedotov Y., Joosten H., Tanneberger F., Moen A. (2017). Mires and peatlands of Europe: Status, distribution and conservation.

[B7565254] Skupinova E. A. (2007). Атлас Вологодской области.

[B7511721] Smirnov N. (2002). Research of environmental group in Shichengskoe Lake. Izvestiya of the Vologda Society for the Study of the Northern Kray.

[B7512425] Sofronova E. V., Abakarova A. S., Afonina O. M., Akatova T. V., Bai X. L., Baisheva E. Z., Bezgodov A. G., Bochkin V. D., Borovichev E. A., Czernyadjeva I. V., Dirksen V. G., Doroshina G. Y., Dulin M. V., Dyachenko A. P., Enkhjargal E., Fedosov V. E., Filin V. R., Ignatov M. S., Ignatova E. A., Ivchenko T. G., Koroteeva T. I., Koryagina E. S., Kotkova V. M., Kuzmina E. Y., Maksimov A. I., Mamontov Y. S., Mežaka A., Nikolajev I. A., Notov A. A., Philippov D. A., Pisarenko O. Y., Potemkin A. D., Sereda V. A., Teleganova V. V., Tsegmed T., Urbanavichene I. I., Urbanavichus G. P., Zheleznova G. V. (2013). New bryophyte records. 2. Arctoa.

[B7512329] Sofronova E. V., Abdurachmanova Z. I., Afonina O. M., Akatova T. V., Andrejeva E. N., Bakalin V. A., Bezgodov A. G., Borovichev E. A., Czernyadjeva I. V., Doroshina G. Y., Dulin M. V., Fedosov V. E., Golovina E. O., Ignatov M. S., Ignatova E. A., Kotkova V. M., Kozhin M. N., Kučera J., Kurbatova L. E., Kushevskaya E. V., Leushina E. G., Makarova M. A., Maksimova T. A., Nikolajev I. A., Philippov D. A., Popova N. N., Potemkin A. D., Prelovskaya E. S., Teleganova V. V., Vilnet A. A., Volkova E. M., Zolotukhin N. I. (2015). New bryophyte records. 5. Arctoa.

[B7512206] Sofronova E. V., Afonina O. M., Aznabaeva S. M., Baisheva E. Z., Bersanova A. N., Bezgodov A. G., Borovichev E. A., Boychuk M. A., Chemeris E. V., Doroshina G. Y., Dulin M. V., Dyachenko A. P., Fedosov V. E., Filippov I. V., Garin E. V., Grishutkin O. G., Ignatov M. S., Ignatova E. A., Ivanova E. I., Kolesnikova M. A., Koroteeva T. I., Kukurichkin G. M., Kutenkov S. A., Kuzmina E. Y., Lapshina E. D., Lavrinenko O. V., Maksimov A. I., Pechenkina K. O., Philippov D. A., Pisarenko O. Y., Popova N. N., Sergeeva Y. M., Shchipanova E. A., Taran G. S., Teleganova V. V., Zakharchenko D. A. (2018). New bryophyte records. 10. Arctoa.

[B7512091] Sofronova E. V., Afonina O. M., Antipin V. K., Belkina O. A., Boychuk M. A., Czernyadjeva I. V., Doroshina G. Y., Dyachenko A. P., Fedosov V. E., Ignatov M. S., Ignatova E. A., Kholod S. S., Kolesnikova M. A., Koltysheva D. E., Komarova A. S., Konstantinova N. A., Koroleva N. E., Koroteeva T. I., Kozhin M. N., Kudr E. V., Kuzmina E. Y., Lavrentiev M. V., Mamontov Y. S., Neshataeva V. Y., Philippov D. A., Popov S. Y., Popova N. N., Sergeeva Y. M., Shevchenko N. E., Smagin V. A., Taran G. S., Teleganova V. V., Teplov K. U., Tikhomirov N. P., Voronkova T. V., Zakharova A. G. (2019). New bryophyte records. 13. Arctoa.

[B7512501] Sterlyagova I. N., Shabalina Y. N., Philippov D. A. (2016). Materials for the algoflora of the Shichengskoe mire (Vologda Region) (Vologda Region).

[B7517058] Stroynov Y. V., Philippov D. A. (2017). Bacterio- and virioplankton in water bodies of a raised bog (Vologda oblast, Russia). Inland Water Biology.

[B7517067] Stroynov Y. V., Philippov D. A. (2017). Virio- and bacterioplankton in primary lakes of the Shichengskoe mire (Vologda Region, Russia). Transactions of Papanin Institute for Biology of Inland Waters RAS.

[B7517140] Suslova T. A., Czhobadze A. B., Philippov D. A., Shiryaeva O. S., Levashov A. N. (2013). A second edition of the Red Data Book of the Vologda Region: revisions in the lists of protected and biological control required species of plants and fungi. Phytodiversity of Eastern Europe.

[B7517087] Udodenko Y. G., Philippov D. A. (2017). Mercury in peat deposits of the Shichengskoe mire (Vologda Region, Russia). Transactions of Papanin Institute for Biology of Inland Waters RAS.

[B7511681] Vishnyakov V. S., Philippov D. A. (2018). New records of charophytes (Charales) from the Northern European Russia. Botanicheskii Zhurnal.

[B7512069] Vorobyev G. A., Korobeynikova L. A., Lyapkina A. A., Lyapkina A. A., Shevelev N. N. (1981). Lake resources of the Vologda region.

[B7565296] Vorobyev G. A. (2007). Природа Вологодской области.

[B7513161] Vørs N. (1992). Heterotrophic Amoebae, flagellates and Heliozoa from the Tvärminne Area, Gulf of Finland, in 1988–1990. Ophelia.

[B7512993] Wieczorek J., Bloom D., Guralnick R., Blum S., Döring M., Giovanni R., Robertson T., Vieglais D. (2012). Darwin Core: An evolving community-developed biodiversity data standard. PLOS One.

[B7518143] Zaytseva V. L., Philippov D. A., Lobunicheva E. V., Mikhaylova A. A. (2014). Influence of *Utriculariaintermedia* on the aquatic invertebrate community structure in mire water tracks. Izvestiya of Samara Scientific Centre of Russian Academy of Sciences.

[B7511671] Zaytseva V. L., Philippov D. A., Lobunicheva E. V. (2016). Zooplankton of raised bogs hollows in the central part of the Vologda Region. Vestnik of Saint Petersburg University. Biology.

[B7511661] Zaytseva V. L., Philippov D. A., Lobunicheva E. V. (2017). Composition and seasonal dynamics of zooplankton in a raised bog stream. Proceedings of Petrozavodsk State University.

